# Monoclinic 122-Type BaIr_2_Ge_2_ with a Channel Framework: A Structural Connection between Clathrate and Layered Compounds

**DOI:** 10.3390/ma10070818

**Published:** 2017-07-18

**Authors:** Xin Gui, Tay-Rong Chang, Tai Kong, Max T. Pan, Robert J. Cava, Weiwei Xie

**Affiliations:** 1 Department of Chemistry, Louisiana State University, Baton Rouge, LA 70803, USA; xgui2@lsu.edu (X.G.); maxpan172910@gmail.com (M.T.P.); 2 Department of Physics, National Cheng Kung University, Tainan 70101, Taiwan; u32trc00@phys.ncku.edu.tw; 3 Department of Chemistry, Princeton University, Princeton, NJ 08540, USA; taik@princeton.edu (T.K.); rcava@princeton.edu (R.J.C.)

**Keywords:** new 122-phase, BaIr_2_Ge_2_, metal-insulator transition (MIT)

## Abstract

A new 122-type phase, monoclinic BaIr_2_Ge_2_ is successfully synthesized by arc melting; X-ray diffraction and scanning electron microscopy are used to purify the phase and determine its crystal structure. BaIr_2_Ge_2_ adopts a clathrate-like channel framework structure of the monoclinic BaRh_2_Si_2_-type, with space group *P*2_1_/*c*. Structural comparisons of clathrate, ThCr_2_Si_2_, CaBe_2_Ge_2_, and BaRh_2_Si_2_ structure types indicate that BaIr_2_Ge_2_ can be considered as an intermediate between clathrate and layered compounds. Magnetic measurements show it to be diamagnetic and non-superconducting down to 1.8 K. Different from many layered or clathrate compounds, monoclinic BaIr_2_Ge_2_ displays a metallic resistivity. Electronic structure calculations performed for BaIr_2_Ge_2_ support its observed structural stability and physical properties.

## 1. Introduction

Quasi-two-dimensional (2D) layered intermetallic compounds attract broad interest in condensed matter physics and solid state chemistry for their various physical and structural properties. Some of the most well-known examples are Fe-based superconducting families, such as FeSe_1−*x*_ [1,2], LaFeAsO_1−*x*_F*_x_* [3], and Ba_1−*x*_K*_x_*Fe_2_As_2_ [4]. In addition to the high temperature superconductors, layered compounds host other strong quantum thermal and spin fluctuations, for example, charge-density-waves (CDWs) [5] and spin-density-waves (SDWs) [6]. Structurally, many two-dimensional layered intermetallics, especially of the 122-type, can be traced back to the parent structure, body-centered tetragonal BaAl_4_ (space group *I*4/*mmm*) [7,8]. In BaAl_4_, the Al atoms, on two independent crystallographic (4*d* and 4*e*) sites, form Al@Al_4_ tetrahedral layers separated by Ba atoms. Derived from BaAl_4_, two major ternary intermetallic families are the ThCr_2_Si_2_ and CaBe_2_Ge_2_-types, with 4*d* and 4*e* sites occupied by transition metals (T) and metalloids (M) [9,10]. Identical by symmetry through the body centering, the ThCr_2_Si_2_ structure contains two equivalent T_2_M_2_ layers per cell. Not only are high T_c_ Fe-based superconductors known in this structure, but strongly correlated electron behavior and magnetic ordering transitions tuned by chemical and physical pressure are also observed [11]. In contrast to ThCr_2_Si_2_, the primitive tetragonal CaBe_2_Ge_2_ structure consists of alternating T_2_M_2_ and M_2_T_2_ layers. Currently, no high T*_c_* superconductors have been reported in the CaBe_2_Ge_2_ structure; but many exotic properties are found for materials in this structure, such as multiple bands leading to the coexistence of charge density waves and superconductivity in SrPt_2_As_2_ [12]. By removing the inversion center from the BaAl_4_ structure, non-centrosymmetric CeCoGe_3_ and CePt_3_Si form, for example [13,14], offering hosts to study superconductivity in non-centrosymmetric structures. Finally, clathrates, which form in different, related structure types, are based on frameworks made primarily of Si, Ge, or Sn (with some M included) with the large atoms are found within the framework cages and are generally known to be semiconducting thermoelectrics, although a small number are known to be superconducting [15,16,17].

The chemical stabilities of many 122-type AT_2_M_2_ compounds can be interpreted using Zintl-Klemm concepts [18]. In these compounds, the polyanion T_2_M_2_ layers and intermediary cation layers alternate along the stacking (c) axis. From the chemical perspective, the differences in the electronegativity of the layers determine whether insulating, semiconducting, semimetallic, or metallic behavior is observed [19]. Here, we report our recent discovery of the 122-phase BaIr_2_Ge_2_. The clathrate-like channel framework of BaIr_2_Ge_2_ can be regarded as an intermediate structure between clathrate and the normally layered compounds with 122 stoichiometry. The new structural motif for a heavy metal 122 germanide offers a new platform to study structure-property relationships in such compounds. The crystal structure, basic electronic and magnetic properties, and calculated electronic structure are presented in the following.

## 2. Experimental

### 2.1. Synthesis of Monoclinic BaIr_2_Ge_2_

The synthesis of BaIr_2_Ge_2_ was performed by arc melting methods, similar to that used for the synthesis of BaIrGe_3_ [20]. Starting materials were barium (>99%, rod, Alfa Aesar, Ward Hill, MA, USA), iridium (99.9%, powder, ~325 mesh, Alfa Aesar) and germanium (99.9999%, pieces, Alfa Aesar). The Ir and Ge were weighed in a 1:1 atomic ratio and were arc-melted together under a high purity, Zr-gettered, argon atmosphere. The monoclinic BaIr_2_Ge_2_ phase was obtained by arc-melting the shiny IrGe droplet and Ba pieces (at 50% excess). BaIr_2_Ge_2_ was stored in the glove box due to its sensitivity to both air and moisture. To investigate the phase stability at different temperatures, we put the as-cast samples into an alumina crucible, which were subsequently sealed in an evacuated (10^-5^ torr) quartz tube and were annealed at 800 °C or 1000 °C for four days. After annealing, the BaIr_2_Ge_2_ compound decomposed to IrGe and unidentified phases.

### 2.2. Phase Identification

**Figure 1:**
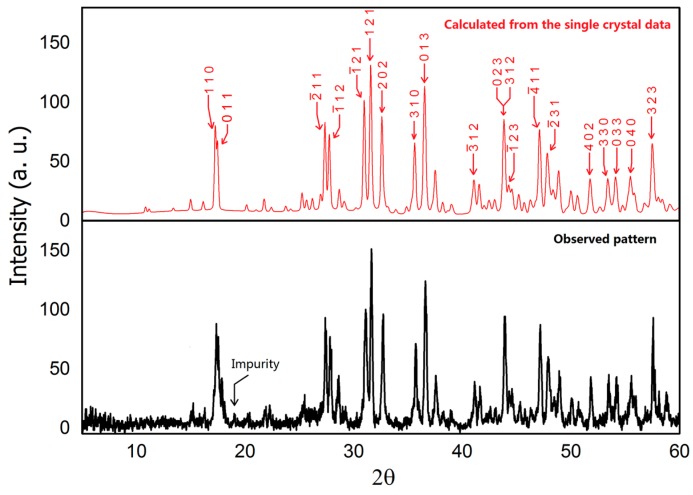
Powder X-ray diffraction pattern of BaIr_2_Ge_2_ (Cu Kα radiation, 300 K). Lower—observed pattern; Upper—calculated pattern with marked Miller indices (hkl) based on the single crystal structure.

Powder X-ray diffraction data was collected using a Rigaku MiniFlex 600 powder X-ray diffractometer (Rigaku, Tokyo, Japan) equipped with Cu K_α_ radiation (λ = 1.5406 Å, Ge monochromator). A Bragg angle 2θ ranging from 5° to 60° with a 0.01° step with a fast scanning mode was employed due to the air-sensitivity of BaIr_2_Ge_2_. The patterns were analyzed using the LeBail method with Jana2006 [21]. (Lower panel in Figure 1) The calculated pattern in Figure 1 (upper panel) was generated using the crystal structure determined from the single crystal X-ray diffraction results. 

### 2.3. Single Crystal Structure Determination

More than five small single crystals (~0.01 × 0.01 × 0.05 mm^3^) from the arc-melted samples of BaIr_2_Ge_2_ were tested to probe the homogeneity of the new phase. A Bruker Apex II diffractometer (Bruker, Billerica, MA, USA) with Mo radiation (λ_Kα_ = 0.71073 Å) was utilized to analyze the sample. The single crystals protected with glycerol were mounted on a Kapton loop (MiTeGen, New York, NY, USA) and scanned with a 2θ range of 5–65° at room temperature. The exposure time was set as 10 s per frame, and the width of scans was 0.5°. To solve the crystal structure, we used direct methods and full-matrix least-squares on F^2^ within the SHELXTL package [22]. Bruker SMART software was applied to make data acquisition, intensity extraction, and corrections for Lorentz and polarization effects [23].

### 2.4. Magnetic Property Measurements

A quantum design physical property measurement system (PPMS) Dynacool (Quantum Design, San Diego, CA, USA) was used to measure the basic magnetic and electronic properties of BaIr_2_Ge_2_. A range from 0 T to 9 T magnetic field at 1.8 K was applied to obtain the field-dependent magnetization. Temperature-dependent magnetization measurements were performed under a magnetic field of 5 T. The 4-probe, zero-field, resistance measurements were carried out in the temperature range from 1.8 K to 300 K.

### 2.5. Electronic Structure Calculations

Crystal orbital hamilton population (COHP) calculations using the tight-binding linear-muffin-tin-orbital (TB-LMTO) method were performed to analyze the atomic interactions in BaIr_2_Ge_2_ and its chemical stability. The *k*-point mesh in the Brillouin zone was set up as 7 × 8 × 7 to perform the calculations. The electronic structures including the density of states (DOS) and the band structure of BaIr_2_Ge_2_ were calculated using the Vienna Ab initio Simulation Package (VASP) based on density functional theory (DFT) [24] with the use of the generalized gradient approximation (GGA) [25]. Spin-orbit coupling (SOC) was included for all the atoms. The cutoff energy was set at 500 eV. A 7 × 8 × 7 Monkhorst-Pack *k*-point mesh with the linear tetrahedron method was used to perform the calculations. The convergence criterion was set to less than 0.1 meV per atom.

## 3. Results and Discussion

According to previous research, most 122-type compounds involving alkali-earth metals, group 14 elements, and cobalt group elements (Co/Rh/Ir) adopt the body-centered tetragonal ThCr_2_Si_2_-type [18,26,27,28,29,30,31,32,33]. However, only two reported compounds, BaRh_2_Si_2_ and BaIr_2_Si_2_, crystallize in BaRh_2_Si_2_-type structure [34]. Different from CaBe_2_Ge_2_ and ThCr_2_Si_2_, which are of the tetragonal layered unit cell, BaRh_2_Si_2_ structure belongs to the monoclinic system. Our synthetic exploration of BaIr_2_Ge_2_ has BaRh_2_Si_2_ structure type according to the single crystal X-ray diffraction. Their crystal structures will be discussed in a subsequent section.

### 3.1. Phase Identification and Structure Determination of BaIr_2_Ge_2_

**Figure 2:**
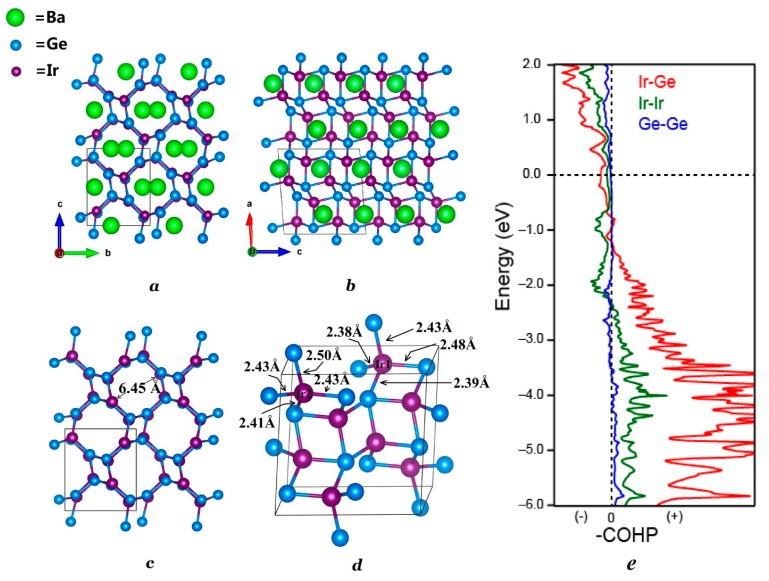
Crystal structure of monoclinic BaIr_2_Ge_2_ refined by single crystal X-ray diffraction. (**a**) View down the a-axis; (**b**) View down the b-axis; (**c**) The IrGe framework. The channels running along the a-axis have a channel of diameter ~6.45 Å; (**d**) close-up of the framework structure showing the Ir-Ge bond lengths; (**e**) Crystal orbital hamilton populations (-COHP) calculation emphasis on the Ir-Ge, Ir-Ir, and Ge-Ge interactions.

The existence of the new BaIr_2_Ge_2_ phase was first seen through the analysis of the powder X-ray diffraction data. Single crystal X-ray diffraction was then used to determine the chemical structure and compositions of BaIr_2_Ge_2_. The crystal structure determined is similar to that of BaRh_2_Si_2_. The powder XRD pattern was successfully indexed and refined using the crystal structure obtained from single crystal XRD. The refined lattice parameters of BaIr_2_Ge_2_ are slightly larger than the ones observed in BaIr_2_Si_2_, which is reasonable due to the atomic radius difference between Si and Ge. The results of the diffraction investigation are summarized in Table 1 and Table 2 which include the atomic positions, site occupancies, and isotropic thermal displacements. Results for refinements using anisotropic thermal displacements are summarized in Table S2 of the Supporting Information. The BaIr_2_Ge_2_ structure crystallizes in the primitive monoclinic space group *P*2_1_/*c* (No. 14) with 20 atoms per unit cell distributed among five crystallographic sites in each unit cell. Mixed site occupancy models have been tested to show whether the atomic distribution in BaIr_2_Ge_2_ is ordered and stoichiometric. The crystal structure, shown in Figure 2, is based on an Ir-Ge channel filled with Ba atoms. The view along the a-axis, illustrated in Figure 2a, emphasizes the Ba-filled Ir-Ge framework. Each of the Ir atoms is surrounded by four Ge atoms, forming irregular tetrahedra; similarly, Ge atoms are surrounded by four Ir atoms. The Ir-Ge distances range from 2.40 Å to 2.50 Å. The Ir@Ge_4_ and Ge@Ir_4_ clusters share edges and form the channel along the *a*-axis. The diameter of the columnar channel is approximately 6.45 Å, which is sufficient for hosting some small chemical molecules (with the Ba removed), such as carbon dioxide and methane.

### 3.2. Structural Comparison of the Different 122 Phases 

The results of the analysis of the bonding interactions using the crystal orbital hamilton population (COHP) method are shown in Figure 2e. The atomic interactions between Ir and Ge in the Ir-Ge polyanion framework dominate the atomic interactions in BaIr_2_Ge_2_. The Fermi level is located in the non-bonding parts in the COHP, which indicates that the structure of BaIr_2_Ge_2_ is electronically stable.

The combination of alkaline-earth elements, a group of 14 metalloids, and Co group metals (Co/Rh/Ir) in a 122 atomic ratio yields three 122-type phases—the BaRh_2_Si_2_-type (Pearson Symbol, *mP*20), the ThCr_2_Si_2_-type (Pearson Symbol, *tI*10), and the CaBe_2_Ge_2_-type (Pearson Symbol, *tP*10). To estimate the structural preferences, the total energies of BaIr_2_Ge_2_ in different 122-type structures were calculated using WIEN2k codes. According to calculations for the total energies of these structures, the BaRh_2_Si_2_-type gives the lowest energy for BaIr_2_Ge_2_, which agrees with our experimental observations.

### 3.3. Structural Connections between Clathrate and Layered Compounds 

**Figure 3:**
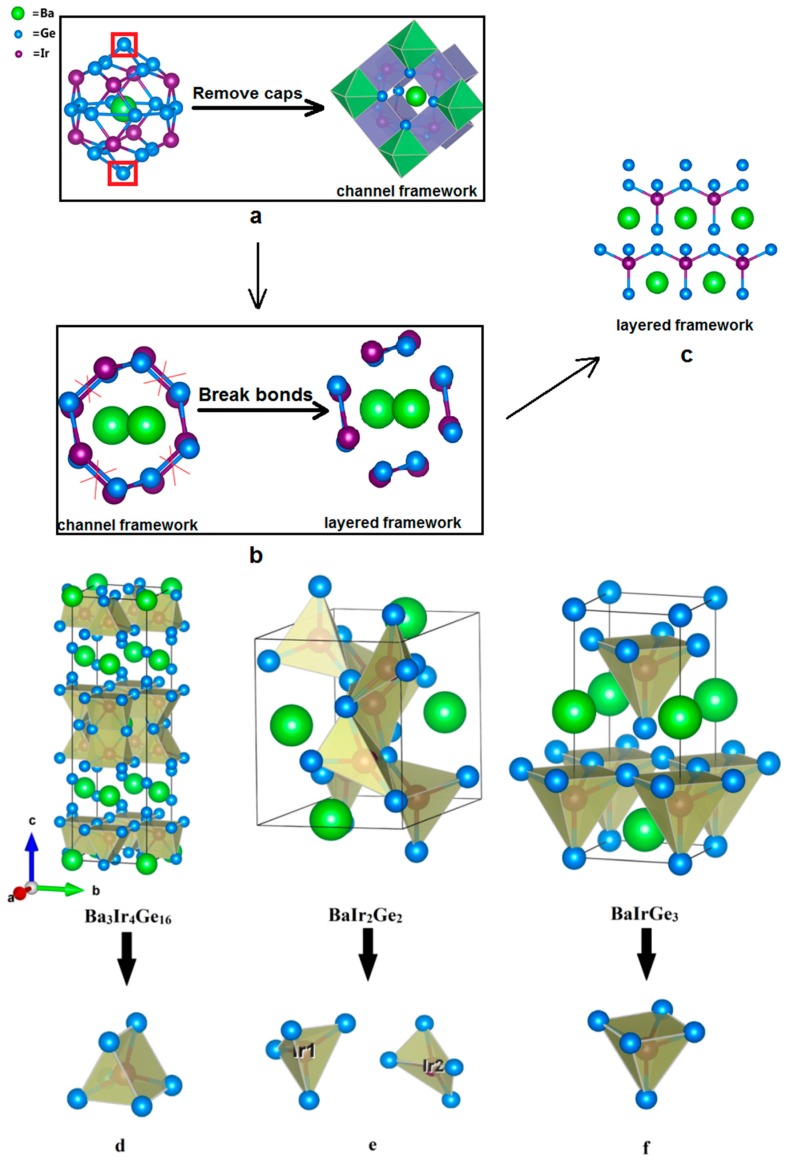
Structural comparison of 122-type phases. (**a**) Clathrate structure of Ba_3_Ir_4_Ge_16_; (**b**) Channel framework of BaIr_2_Ge_2_; (**c**) Layered structure of BaIrGe_3_; (**d**) The symmetrical pentahedron in Ba_3_Ir_4_Ge_16_; (**e**) The irregular, non-symmetrical tetrahedron in BaIr_2_Ge_2_; (**f**) The square pyramid in BaIrGe_3_.

The other interesting ternary compounds in the Ba-Ir-Ge system are the body-centered tetragonal superconductor, Ba_3_Ir_4_Ge_16_ [35] and non-centrosymmetric tetragonal BaIrGe_3_ [36]. As shown in Figure 3, Ir-centered square pyramids, symmetrical pentahedra, and irregular, non-symmetrical tetrahedra are formed in BaIrGe_3_, Ba_3_Ir_4_Ge_16_, and BaIr_2_Ge_2_, respectively. The crystal structure of the superconductor Ba_3_Ir_4_Ge_16_ reveals that it contains the unique edge-shared crown-shaped Ba@Ge_16_ polyhedra [35]. The systems with heavy cations “rattling” inside the oversized lattice cavities are called clathrate type structures. Moreover, the open channels around the chains filled with electropositive metals can be considered as clathrate-like structure forms [16]. Accordingly, BaIr_2_Ge_2_ can also be regarded as the clathrate-like compound with Ba rattling inside the open chains, which consist of edge-sharing Ir@Ge_4_ irregular tetrahedra in Figure 3. One can easily see that BaIrGe_3_ is a layered compound with Ir centered in the vertex-sharing Ge square pyramids along the *ab*-plane. Therefore, the clathrate-like BaIr_2_Ge_2_ structure is likely an intermediate structure between regular clathrate and layered structures. 

### 3.4. Magnetic Properties of Monoclinic BaIr_2_Ge_2_

**Figure 4:**
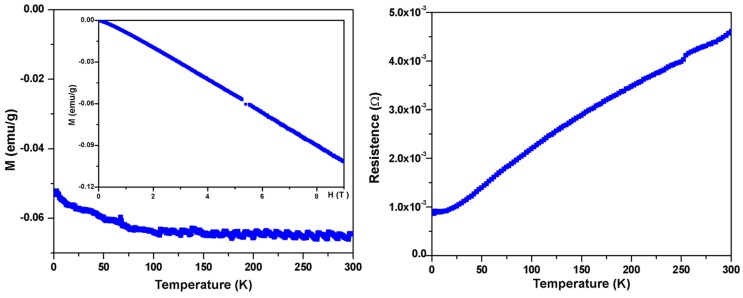
Basic electronic and magnetic characterization of BaIr_2_Ge_2_. (**Left:** Main Panel) Temperature-dependent magnetic susceptibility (M/μ_0_H at μ_0_H = 5 T) and (**Left:** Inserted) field-dependent magnetization measurements showing the near linearity of M (magnetization) vs. H (magnetic field) for fields beyond 5 T at 1.8 K, justifying the use of the a high applied field in the measurements; (**Right**) Zero-field resistance data from 1.8 K to 300 K.

To further study the physical properties of BaIr_2_Ge_2_, magnetic measurements were carried out. First, no superconductivity was observed above 1.8 K (in low applied field (20 Oe) measurements). Moreover, the relatively small temperature-independent molar magnetic susceptibility, presented in Figure 4 (Left, Main Panel), is dominated by core diamagnetism; the magnetic susceptibility of the material is around −6 × 10^−2^ emu/g. The magnetic isotherm measurements in Figure 4 (Left, Inserted) showing diamagnetic behavior at 1.8 K are in agreement with the temperature-dependent magnetization. This indicates that the paramagnetic contribution of conduction electrons to the observed susceptibility is small, which is indirect evidence to support the Zintl-like characteristics of BaIr_2_Ge_2_. The resistance measurements in Figure 4 (Right) show a metallic behavior with a residual resistance ratio of ~5.

### 3.5. Electronic Structure Calculations

**Figure 5:**
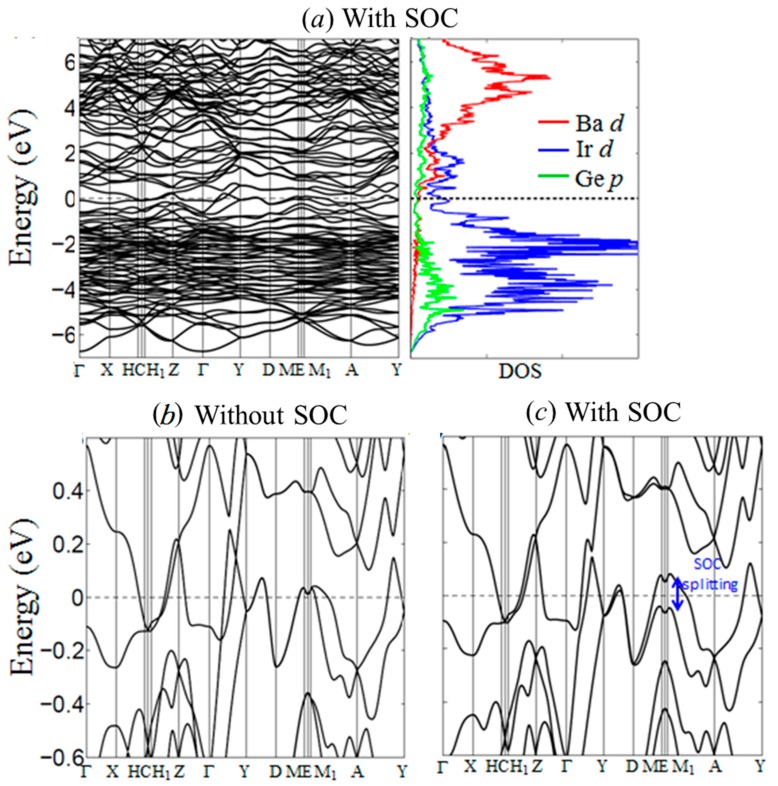
Calculated electronic band structure and density of states (DOS) of BaIr_2_Ge_2_ using generalized gradient approximation (GGA). (**a**) Band structure and DOS with spin-orbit coupling emphasis on the energy range from −7 to +7 eV; (**b**) Band structure calculated by GGA without spin-orbit coupling; (**c**) Band structure calculated by GGA with spin-orbit coupling.

Electronic calculations were carried out to evaluate and analyze the electronic density of states (DOS) and band structure, which can help provide a better understanding of the structural stability and physical properties of BaIr_2_Ge_2_. The partial DOS curves (Figure 5a) emphasize the contributions from the valence orbitals of each atom and show that the Fermi level (E_F_) lies in a broad pseudo gap in the DOS. This is indicative of the chemical stability of the compound, and also, due to the low density of states, is consistent with the diamagnetism and metallic resistivity observed (see below); no saddle point is seen in the band structure in the vicinity of the Fermi level, making the lack of observed superconductivity above 1.8 K consistent (again, see below) with some ideas for what gives rise to superconductivity in many compounds [37]. Based on the comparison of the electronic structure results with and without the inclusion of spin-orbit coupling, the major impact of the SOC near the E_F_ is a band splitting into states derived primarily from the 6*s*/5*d* orbitals of the Ir atoms, see Figure 5d. We speculate that doping with electronegative elements like P or A may induce a metal-insulator transition (MIT) in BaIr_2_Ge_2_. 

## 4. Conclusions

The new BaIr_2_Ge_2_ compound, with monoclinic structure *P*2_1_/*c* (S.G.14), was successfully synthesized using arc-melting. The crystal structure of BaIr_2_Ge_2_ shows a clathrate-like channel framework of Ir-Ge, which can be regarded as an intermediate structure between clathrate and layered compounds. Electronic structure calculations and chemical bonding interactions of BaIr_2_Ge_2_ were investigated to help understand the chemical stability of the compound and its properties. Magnetic measurements indicate diamagnetic susceptibility of BaIr_2_Ge_2_ without the observation of superconductivity down to 1.8 K. The resistivity measurements indicate metallic properties for BaIr_2_Ge_2_, and electronic structure calculations are consistent with the observed behavior. Future efforts will be focused on inducing a metal-insulator transition in BaIr_2_Ge_2_. 

## Figures and Tables

**Table 1:** Single crystal crystallographic data for BaIr_2_Ge_2_ at 299 (2) K.

Refined Formula	BaIr_2_Ge_2_
Formula weight (F.W.) (g/mol)	666.92
Space group; Z	*P*2_1_/*c* (No. 14); 4
*a* (Å)	8.204 (5)
*b* (Å)	6.625 (4)
*c* (Å)	7.959 (5)
β (°)	94.27 (1)
V (Å^3^)	431.4 (4)
Extinction Coefficient	0.00061 (9)
θ range (deg)	2.489–32.085
*hkl* ranges	−12 ≤ *h* ≤ 12
	−9 ≤ *k* ≤ 9
	−11 ≤ *l* ≤ 10
No. reflections; *R_int_*	9731; 0.0925
No. independent reflections	1483
No. parameters	47
*R*_1_: *ωR*_2_ (all *I*)	0.0503; 0.0872
Goodness of fit	0.954
Diffraction peak and hole (e^−^/Å^3^)	3.812; −3.705

**Table 2:** Atomic coordinates and equivalent isotropic displacement parameters for BaIr_2_Ge_2_ in space group *P*2_1_/*c*. U_eq_ is defined as one-third of the trace of the orthogonalized U_ij_ tensor (Å^2^).

Atom	Wyckoff.	Occ.	*x*	*y*	*z*	*U_eq_*
Ba1	4*e*	1	0.2318 (1)	0.8818 (2)	0.4993 (2)	0.0116 (3)
Ir2	4*e*	1	0.6260 (1)	0.8959 (1)	0.1069 (1)	0.0074 (2)
Ir3	4*e*	1	0.8532 (1)	0.6648 (1)	0.3334 (1)	0.0077 (2)
Ge4	4*e*	1	0.5560 (2)	0.8515 (3)	0.8069 (3)	0.0088 (4)
Ge5	4*e*	1	0.9282 (2)	0.9171 (3)	0.1356 (3)	0.0091 (4)

## Supplementary Materials

The following are available online at www.mdpi.com/1996-1944/10/7/818/s1, Table S1: SEM-EDX, Table S2: Anisotropic Thermal Parameters of BaIr_2_Ge_2_ from single crystal diffraction.
