# Supplementary material for: Monoclinic 122-Type BaIr2Ge2 with a Channel Framework: A Structural Connection between Clathrate and Layered Compounds
Source: Materials (Basel). 2017 Jul 18;10(7):818. doi: 10.3390/ma10070818 (PMC5551861; doi:10.3390/ma10070818)
Supplement: Supplementary file 1 [file materials-10-00818-s001.zip › SI-BaIr2Ge2(1).docx]

**New Monoclinic 122-type BaIr_2_Ge_2_ with Channel Framework: A Transition from Clathrate to Layered Compounds**

Xin Gui ^1^, Tay-Rong Chang ^2^, Tai Kong ^3^, Max T. Pan ^1^, Robert J. Cava ^3^ and Weiwei Xie ^1,^*

^1^ Department of Chemistry, Louisiana State University, Baton Rouge, LA 70803, USA; xgui2@lsu.edu (X.G.); maxpan172910@gmail.com (M.T.P.)

^2^ Department of Physics, National Cheng Kung University, Tainan 70101, Taiwan; u32trc00@phys.ncku.edu.tw

^3^ Department of Chemistry, Princeton University, Princeton, NJ 08540, USA; taik@princeton.edu (T.K.); rcava@princeton.edu (R.J.C.)

***** Correspondence: weiweix@lsu.edu; Tel.: +1-225-578-1074

**Supplementary information**

**Table S1.** Stoichiometric ratio for BaIr_2_Ge_2_ tested by Scanning Electron Microscope-Energy-dispersive X-ray spectroscopy (SEM-EDS)

| **Element** | **Weight %** | **Atomic %** | **Net Int.** | **Error %** | **K ratio** | **Z** | **R** | **A** | **F** |
| --- | --- | --- | --- | --- | --- | --- | --- | --- | --- |
| Ge | 26.46 | 45.76 | 843.30 | 5.26 | 0.1936 | 1.1444 | 0.8860 | 0.6419 | 0.9962 |
| Ir | 49.76 | 32.50 | 1587.75 | 3.13 | 0.4144 | 0.9310 | 1.0781 | 0.8721 | 1.0257 |
| Ba | 23.78 | 21.74 | 386.06 | 4.55 | 0.2241 | 0.9913 | 1.0054 | 0.9429 | 1.0080 |

**Table S2.** Anisotropic thermal displacements from BaIr_2_Ge_2_

| Atom | U11 | U22 | U33 | U23 | U13 | U12 |
| --- | --- | --- | --- | --- | --- | --- |
| Ba3 | 0.0133(5) | 0.0100(6) | 0.0112(6) | -0.0003(5) | -0.0010(4) | -0.0002(5) |
| Ir1  Ir2 | 0.0082(3)  0.0083(3) | 0.0070(4)  0.0071(4) | 0.0069(4)  0.0076(4) | 0.0003(3)  0.0007(3) | 0.0002(2)  -0.0008(3) | 0.0006(3)  0.0003(3) |
| Ge4 | 0.0083(9) | 0.0097(12) | 0.0082(10) | -0.0008(9) | -0.0002(7) | 0.0012(8) |
| Ge5 | 0.0066(9) | 0.0097(11) | 0.0108(11) | 0.0006(9) | -0.0009(7) | -0.0003(8) |
